# Design and Fabrication of Optical Flow Cell for Multiplex Detection of β-lactamase in Microchannels

**DOI:** 10.3390/mi11040385

**Published:** 2020-04-05

**Authors:** Sammer-ul Hassan, Xunli Zhang

**Affiliations:** 1 Bioengineering Research Group, Faculty of Engineering and Physical Sciences, University of Southampton, Southampton SO17 1BJ, UK; 2 Institute for Life Sciences, University of Southampton, Southampton SO17 1BJ, UK

**Keywords:** microfluidics, point-of-care (POC) diagnostics, antimicrobial resistance (AMR), lab-on-a-chip, absorbance, optical detections, linear actuators, beta-lactamase, analytical chemistry

## Abstract

Miniaturized quantitative assays offer multiplexing capability in a microfluidic device for high-throughput applications such as antimicrobial resistance (AMR) studies. The detection of these multiple microchannels in a single microfluidic device becomes crucial for point-of-care (POC) testing and clinical diagnostics. This paper showcases an optical flow cell for detection of parallel microchannels in a microfluidic chip. The flow cell operates by measuring the light intensity from the microchannels based on Beer-Lambert law in a linearly moving chip. While this platform could be tailored for a wide variety of applications, here we show the design, fabrication and working principle of the device. β-lactamase, an indicator of bacterial resistance to β-lactam antibiotics, especially in milk, is shown as an example. The flow cell has a small footprint and uses low-powered, low-cost components, which makes it ideally suited for use in portable devices that require multiple sample detection in a single chip.

## 1. Introduction

Point-of-care (POC) and clinical diagnostics require state of the art quantitative assays at high throughput and in real-time near the patient. POC diagnostics devices are widely used in the market for certain tests such as urinalysis, pregnancy tests, blood glucose levels, sepsis, prostate and ovarian cancer and cardiovascular diseases. POC devices must be able to detect and quantify the early stages of these diseases/infections [1,2,3]. However, the POC devices have still minimal use for diagnostics in clinical settings due to low sensitivity, complexity and low-throughput. The outlook of the POC devices is to provide quick analysis which can lead towards the quicker clinical decisions and hence allow the early start of treatments for patients. These earlier well-informed treatments will have better chances to improve the health of the patients as compared to the less-informed treatments while waiting for the central lab results [4,5].

The requirement to perform multiplex diagnostics in a single POC device has led to the development of multiple microchannel systems capable of loading multiple samples [6]. With the advancement of the innovative technologies, multiplexing samples in a microfluidic chip has become practical. While the development of multiple sample lanes on a single chip has been geared up but the requirement of high-throughput and portable detections has not been fully exploited and developed. Multiple detection methods, including electrochemical and optical detection systems, have been developed over the past decade. Electrochemical detection utilizes a very sensitive sensor that detects chemical reactions that take place at its electrode. This detection method can be integrated into microchannels and the electrochemical detection systems are usually more compact than alternative methods such as optical detection [7]. However, electrochemical detection methods have problems, including the electrode sensitivity changing over time [8], though designs have been created with replaceable electrodes [9] and are widely used in the market. Optical detection method, on the other hand, typically uses a light source to illuminate the sample in the microchannel and unlike the electrochemical sensor made of an electrode that is usually embedded inside the channel; the optical detector could move from one channel to another and scan many microchannels in sequence in a short period of time [10]. 

Most widely used optical detection methods include ultraviolet/visible (UV/Vis) absorption spectroscopy, fluorescence, chemiluminescence [7] and smartphone detections [11]. Sensitivities vary between these optical detection methods with fluorescent imaging having higher sensitivity than other optical methods. However, fluorescent detection of microchannels requires expensive optical components and complicated signal controls and are, therefore, not suitable for portable and inexpensive POC diagnostics [12]. Smartphones are also emerging as an alternative detection system for colorimetric assays but the current approaches demand expensive optics and are limited to a few microchannels [11]. Moreover, the variability in smartphone cameras and light intensity distribution amongst microchannels makes them less accurate and hinders their use in clinical settings. 

Absorption spectroscopyis one of the techniques used in microfluidics to quantify analytes of interest, which is described by Beer-Lambert law. Several LEDs could be used in combination with various filters to detect different analytes while the overall instrument cost can be still low [13,14]. Detection of several microchannels requires multiple LED/Photodiode arrays with multiple electrical connections in series. This is impractical due to the variations in sensitivities between detectors, increased costs and bigger size of the device. Our device offers simple operation and detection of multiple microchannels, in a single chip, by simply moving a microchip in a single LED/Photodiode flow cell. The microchannels pass through the detector one by one using a homemade linear actuator and a signal is recorded continuously in 15 microchannels. 

## 2. Experimental

### 2.1. Microchip Fabrication

The microfluidic chip was designed using AutoCAD (Autodesk, Inc., San Rafael, CA, USA) and 8 mm long microchannels were micromilled on a 1.2 mm thick polymethyl methacrylate (PMMA) surface via precision micromilling (0.4 mm width × 0.4 mm height). The milled PMMA piece (10 mm × 13.6 mm: length × Width) was bonded with another PMMA piece of similar dimensions via solvent bonding. Briefly, the PMMA sheets were cleaned with propan-2-ol, air-dried and placed on a glass petri dish. The mixture of ethanol and acetone (50:50) was poured on the surface and left for 20–30 s. The sheets were then combined together and the microchip was left for 30–40 s and dried gently with compressed air. The excess liquid must be removed from the microchannels completely. The microchip was then placed under metal piece (3–4 Kg weight) for 5 min. The microchip was then placed in an oxygen plasma cleaner (Diener electronics, Ebhausen, Germany) for 8 min at a power of 90 watts. The microchannels were filled with 2% polyvinyl alcohol (PVA) (146,000–186,000), left for 10 min at room temperature, air-dried and placed in an oven for 15 min. Then, 2% PVA solution consisting of glutaraldehyde (5 mM) and hydrochloric acid (5 mM) was introduced into the microchannels and left for 2 h at 37 °C. PVA Coated and cross-linked microchannels were loaded with different concentrations of nitrocefin (0.050, 0.075, 0.125 and 0.250 mg/mL) and left for 30 min at room temperature. The microchannels were then air-dried and a β-lactamase solution (0.2 mg/mL) in phosphate buffered saline (PBS) with pH of 7.2 or in milk was injected into the microchannels. 

### 2.2. Fabrication of Flow Cell

**Figure 1:**
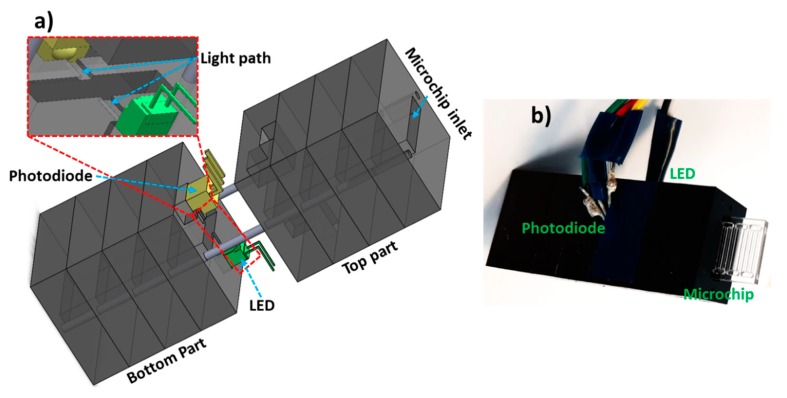
Optical flow cell overview. (**a**) Schematic of the portable flow cell illustrating LED, photodiode, light path and microchip inlet. (**b**) Photograph of the flow cell with a microchip inserted into the inlet. Multiple PMMA pieces (5 mm thickness) were micromilled and combined via alignment pins. Bottom par houses the LED and photodiode while the top part allows the electrical connections to the microcontroller.

Miniaturized flow cell based on Beer-Lambert law was fabricated using precision micromilling of Black sheets of PMMA (5 mm thickness), off-the-shelf LED (Avago Technologies, San José, CA, USA) and a photodiode (TSL257, Texas Advance Optical Solutions, Cambridge, UK) as shown in Figure 1a. LED was powered by 12 V DC power supply at a fixed volts of 2.85 V and the photodiode was powered via 5 V pin at microcontroller (Arduino, CA, USA). Lightpath (0.3 mm width × 0.3 mm height) was created in between the LED and photodiode and a microchip inlet slit (10 mm × 2.4 mm: width × height) was fabricated. The overall dimensions of the flow cell are 40 × 20 × 12.4 mm (length × width × height). The microchip consisting of multiple channels was inserted into the flow cell, as shown in Figure 1b. The transmitted light is collected at the photodiode that sends the signal to the microcontroller (Arduino, CA, USA), which is collected by Arduino software on the PC and further processed by LabView (National Instruments, Austin, TX, USA) [14]. LEDs are replaceable in the flow cell and used in the following experiments based on the absorbance peak requirements. For all the calibrations with red food dye, the LED with peak wavelength of 527 nm was used while for β-lactamase assay, the LED with peak wavelength of 490 nm was used.

### 2.3. Linear Actuator Design and Fabrication

**Figure 2:**
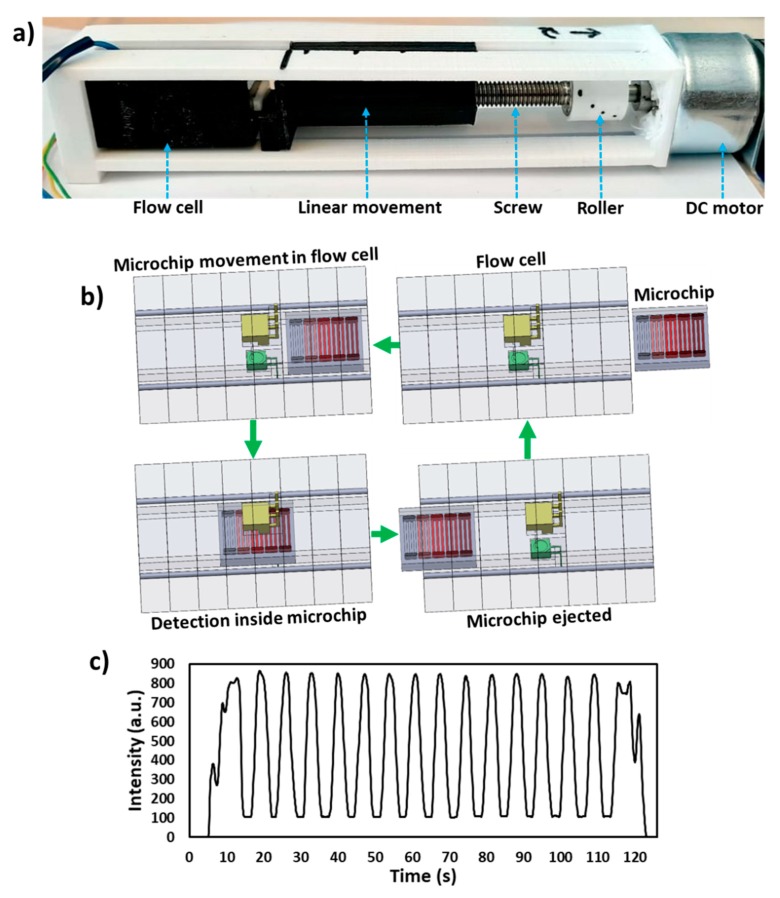
Working principle of the device. (**a**) Photograph of the device illustrating DC motor, roller, screw, nut, a linearly moving push arm, flow cell and a 3D printed casing. (**b**) Schematic of the working principle of the device. The DC motor moves the push arm linearly towards the flow cell, which moves microchip inside the flow cell passing all the microchannels from the detector and a signal is recorded. The microchip is finally ejected from the flow cell. (**c**) Transmitted light intensity profile of the 15 microchannels passed from the flow cell containing red food dye.

The linear actuator design and fabricated components are shown in Figure 2a. The linear actuator consists of a DC motor (RS components, Corby, UK), roller (3D printed), screw, nut, a linearly moving push arm and a 3D printed casing which allows accurate alignment of all the components. A roller was designed with Solidworks (Dassault Systems Solidworks Corp., Waltham, MA, USA), fabricated using 3D printing (Ultimaker 2+, Ultimaker B.V., Utrecht, Belgium) and fixed onto the motor shaft. The screw was placed into the grove fabricated in the roller and was rigid fixed with glue. The nut was glue fixed in the push arm and screwed on the screw. The push arm was designed in a way only to allow linear movement in forwarding and backward directions. The 3D printed polylactic acid (PLA) casing was screw fixed on the motor, as shown in Figure 2a. The flow cell was also placed into the casing with the microchip inlet aligned to the push arm. The overall dimensions of the device including flow cell, motor and linear actuator are 16 × 2.5 × 3 cm (length × width × height). The device dimensions can be further minimized by integrating miniaturized linear actuators, smaller shafts, motors and thinner plastics for portable and compact systems.

### 2.4. Working Principle of the Device

The schematic of the working principle of the device is shown in Figure 2b. A microchip with samples placed in microchannels is placed inside the flow cell and a push arm is aligned with the microchip. The DC motor is switched on (12 V DC power supply) which moves the push arm linearly towards flow cell and hence the microchip moves inside the flow cell (Figure 2b). Each microchannel passes from the light path between the LED and photodiode and transmitted light intensity is recorded via the microcontroller. The 15 microchannels were filled with red food dye (East End Foods plc, West Bromwich, UK) and passed through the flow cell and signal was recorded, as shown in Figure 2c. Finally, the microchip is ejected from the flow cell.

## 3. Results and Discussions

### 3.1. Characterisation of Linear Actuator

**Figure 3:**
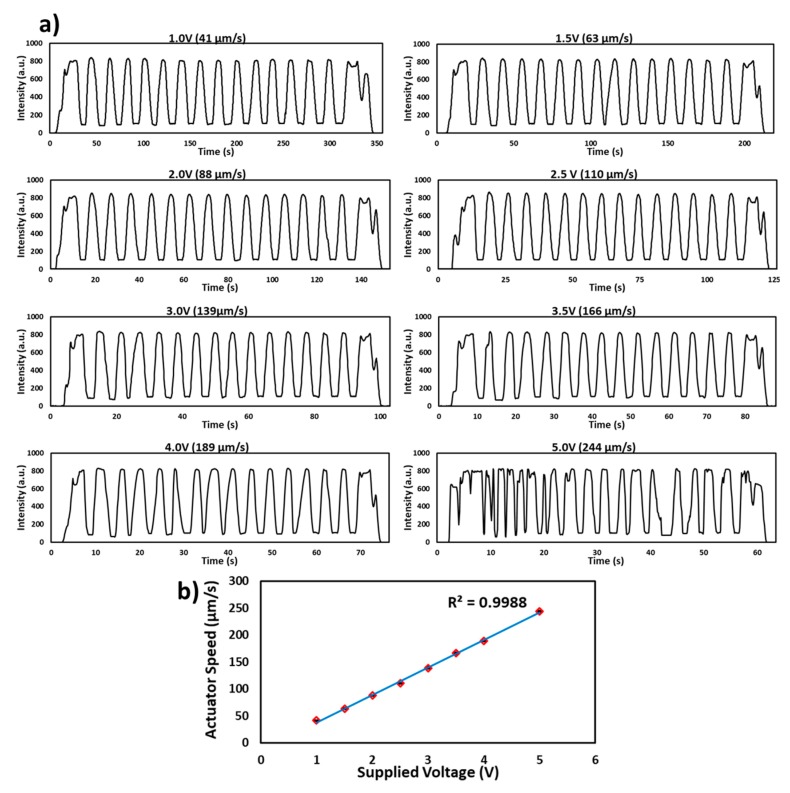
Characterization of the linear actuator. (**a**) Transmitted light intensity profile of the red food dye filled microchip passed through the flow cell at different volts (1.0, 1.5, 2.0, 2.5, 3.0, 3.5, 4.0 and 5.0 V). The signal is stable up to the speed of 189 µm/s at 4.0 V. (**b**) Graph of the supplied voltage to the motor and a speed of the microchip in the flow cell. The speed of the microchip increases linearly with increase in supplied volts.

To test the performance and stability of the linear actuator, a microchip with 15 channels filled with red food dye was inserted into the flow cell. The motor was run at different voltages (1.0, 1.5, 2.0, 2.5, 3.0, 3.5, 4.0 and 5.0 V) and a signal was recorded as shown in Figure 3a. The speed of the linear actuator was measured via noting of time (s) against fixed movement of push arm for each voltage and was recorded as 41, 63, 88, 110, 139, 166, 189 and 244 µm/s). It was found that the movement of the microchip and sample signal was stable up to 4.0 V and an unstable signal was found above this voltage. Therefore, with the current motor and using 15 microchannels, the results can be achieved as quickly as in 70 s. However, the detection time can be improved by using sophisticated/stepper motors. Figure 3b shows a linear relationship between the volts supplied to the motor and the speed of the microchip in the flow cell. 

### 3.2. Characterisation of the Flow Cell

**Figure 4:**
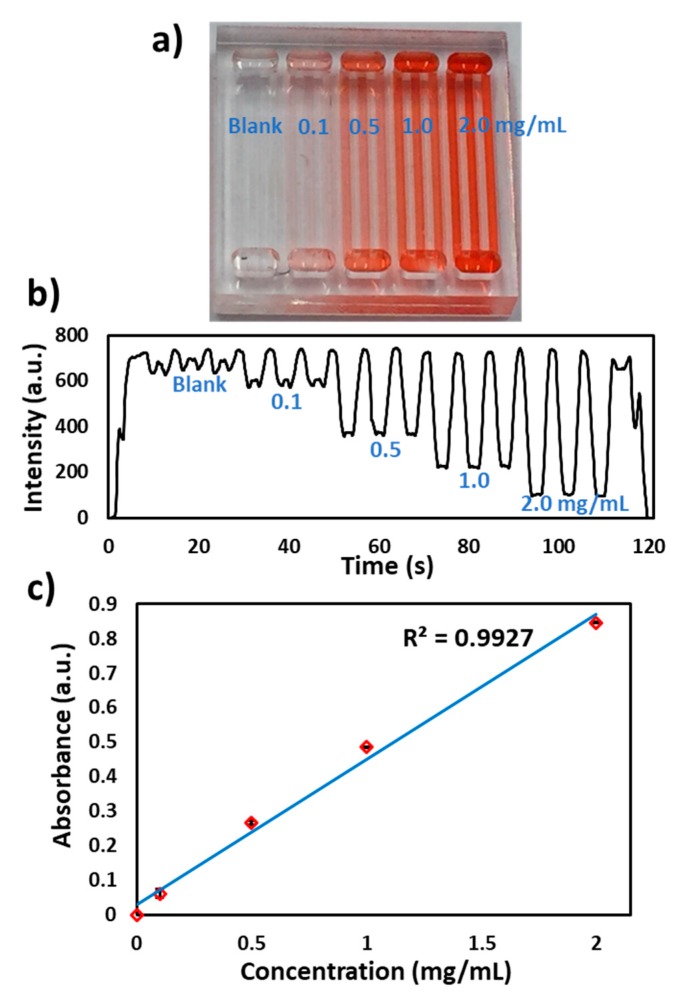
Characterization of the flow cell. (**a**) Photograph of different concentrations of red food dye in microchannels (blank, 0.1, 0.5, 1.0 and 2.0 mg/mL). (**b**) Transmitted light intensity profile of the microchip with different food dye concentrations. (**c**) Absorbance calibration of the detector response for different food dye concentrations. The absorbance of the food dye increases linearly with increase in dye concentration.

To test the performance of the flow cell, red food dye was filled in 15 channels and inserted into the flow cell. The actuator was run at a voltage of 2.5 V (110 µm/s) and a signal was recorded, as shown in Figure 2c. The absorbance from each channel was measured and percent relative standard deviation (%RSD) between each channel was found to be <2%. To calibrate the flow cell, a set of red food dye concentrations (blank, 0.1, 0.5, 1.0 and 2.0 mg/mL) were filled inside the microchip (Figure 4a), inserted into the flow cell and linearly moved at a speed of 110 µm/s. The signal was plotted as light intensity against time, (Figure 4b) and the absorbance was measured against each concentration and a linear curve was obtained (Figure 4c) with variability in each concentration channels <5% (%RSD, n = 3). For the lowest concentrations, the entry and exit of the microchannel from the lightpath is accompanied by a brief spike in the light intensity due to lensing by the microchannel edge. As the concentration increases, this effect becomes less noticeable as it becomes masked by the higher absorbance. Nonetheless, these spiked or unexpected peaks do not affect the transmitted light intensity readings. The speed of the linear actuator, the microchannel width and lightpath are optimized in such a way as to achieve flat signal readings from each channel and hence are used for absorbance measurements. 

### 3.3. β-lactamase Activity in Microchannels

**Figure 5:**
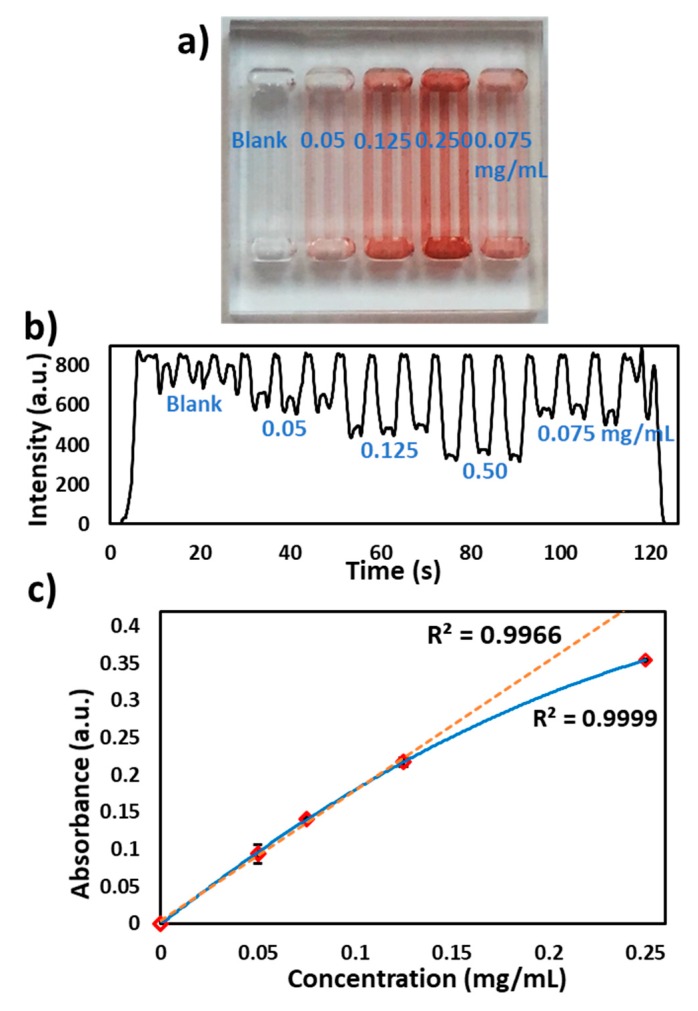
Nitrocefin coated microchip reaction with β-lactamase in microchannels. (**a**) Photograph of the microchip with the loading of different concentrations of nitrocefin (blank, 0.050, 0.075, 0.125 and 0.250 mg/mL), (**b**) Transmitted light intensity profile of reaction of different concentrations of nitrocefin with β-lactamase. (**c**) Graph of the nitrocefin concentration vs absorbance measured via the flow cell.

The presence of antibiotic residues in milk, especially β-lactams are allergic, carcinogenic and cause the development of antibiotic-resistant bacterial strains. β-lactamase presence in milk degrades antibiotics and its resistance has become a global challenge. Rapid detection of antibiotic residues in milk samples is of immense importance to the dairy industry [15,16,17]. Kumar S. et al. [18] developed a chromogenic substrate-based assay for specific detection of the β-lactam group (penicillins, carbapenems) in milk, which works on the principle of a chromogenic β-lactamase substrate (nitrocefin) undergoing a color change from yellow to red. Microchannels were coated with nitrocefin, as previously described by Reis et al. [19] and provided in the methods. A β-lactamase solution (0.2 mg/mL) in PBS (pH 7.2) was injected into the microchannels and a red color was observed (Figure 5a). The microchip was passed through the flow cell at 2.5 V (490 nm LED) and a signal was recorded as shown in Figure 5b. Figure 5c shows the relationship between the absorbance measured from the flow cell and the concentration of the nitrocefin (in multiples of three). The deviation from the linearity can be explained via the intrinsic characteristic of Beer-Lambert law. The nitrocefin coatings were applied to multiple chips (n = 3) and the assay was repeated as discussed above. The absorbance results were found to be reproducible (8%RSD) between the microchips and (7%RSD) within the microchannels. The variations in the absorbance values can be attributed to the differences in the PVA coating and reagents cross-linking inside microchannels.

**Figure 6:**
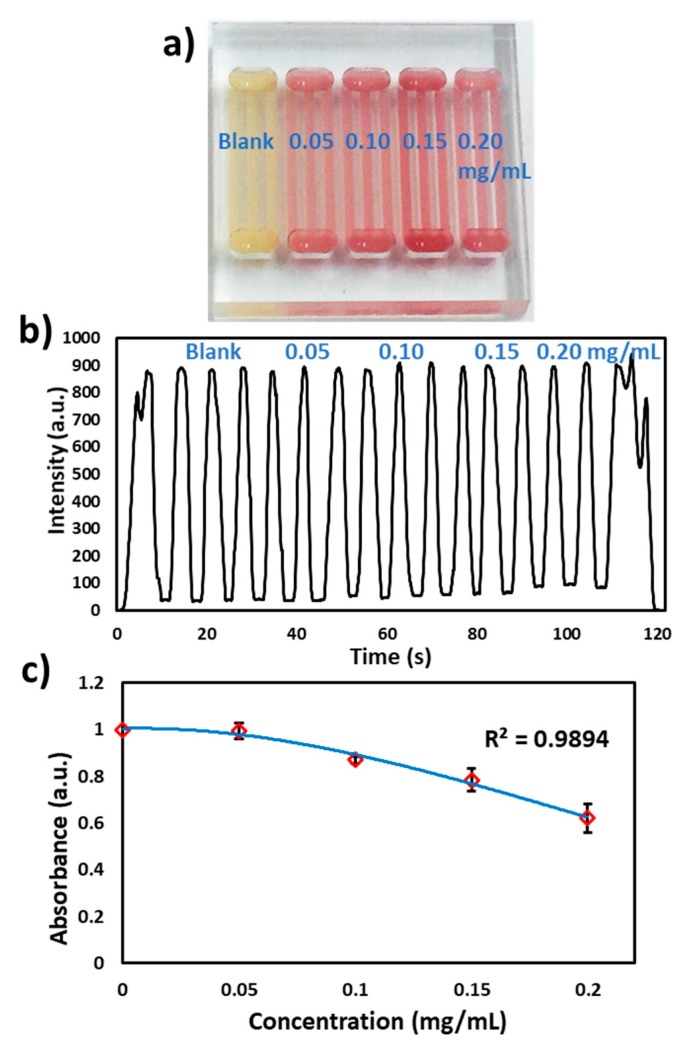
β-lactamase activity in milk. (**a**) Photograph of the microchip with loading of different concentrations of β-lactamase (blank, 0.05, 0.10, 0.15 and 0.20 mg/mL), (**b**) Transmitted light intensity profile of reaction of different concentrations of β-lactamase with nitrocefin. (**c**) Graph of the β-lactamase concentration vs absorbance measured via the flow cell.

Furthermore, nitrocefin (0.5 mg/mL) was coated in the microchannels as discussed above and reacted with different concentrations of β-lactamase spiked in milk (blank, 0.05, 0.10, 0.15 and 0.20 mg/mL) as shown in Figure 6a. The microchip was passed through the flow cell at 2.5 V and the signal was recorded. At 490 nm, the milk absorbance was found to be more than 90% (Figure 6b). However, the flow cell was able to detect the differences in absorbance of each concentration, as shown in Figure 6c.

## 4. Conclusions

The simple-to-operate and portable nature of this flow cell, combined with the ability to analyze multiple sample channels at low-cost promises to revolutionize point-of-care diagnostics, especially tackling antimicrobial resistance in healthcare. This paper showed the design and working principle of an optical flow cell for detection of parallel microchannels in a microfluidic chip. The flow cell operates by measuring the light intensity from the microchannels and a microchip was linearly moved inside the flow cell via homemade linear actuator. We have shown the proof-of-principle of the device along with calibrations and an example of β-lactamase, an indicator of bacterial resistance to β-lactam antibiotics. The flow cell will be further exploited for colorimetric based assays especially spore based assays for β-lactam antibiotics in milk.
